# Combined Analysis of Plasma Amphiregulin and Heregulin Predicts Response to Cetuximab in Metastatic Colorectal Cancer

**DOI:** 10.1371/journal.pone.0143132

**Published:** 2015-11-16

**Authors:** Kimio Yonesaka, Naoki Takegawa, Taroh Satoh, Hiroto Ueda, Takeshi Yoshida, Masayuki Takeda, Toshio Shimizu, Yasutaka Chiba, Isamu Okamoto, Kazuto Nishio, Takao Tamura, Kazuhiko Nakagawa

**Affiliations:** 1 Department of Medical Oncology, Kinki University Faculty of Medicine, Osaka, Japan; 2 Department of Frontier Science for Cancer and Chemotherapy, Osaka University Graduate School of Medicine, Osaka, Japan; 3 Clinical Research Center, Kinki University Faculty of Medicine, Osaka, Japan; 4 Center for Clinical and Translational Research, Kyushu University, Fukuoka, Japan; 5 Department of Genome Biology, Kinki University Faculty of Medicine, Osaka, Japan; RIKEN Center for Integrative Medical Sciences, JAPAN

## Abstract

**Background:**

Amphiregulin, a ligand of the epidermal growth factor receptor (EGFR), is associated with the efficacy of cetuximab, an antibody against EGFR, as treatment for colorectal cancer (CRC). In contrast, the HER3 ligand heregulin correlates with cetuximab resistance. In this study, we evaluated how the combined levels of circulating amphiregulin and heregulin affect clinical outcomes in patients who receive cetuximab as therapy against advanced CRC.

**Methods:**

Plasma levels of amphiregulin and heregulin were measured by enzyme-linked immunosorbent assay in 50 patients with CRC in a training cohort, and in 10 patients in a validation cohort. The combined expression was then assessed with clinical outcome after receiver operating characteristics analysis.

**Results:**

Overall response rate was 26%, and median progression-free survival was 110 days in the training cohort. Patients with high amphiregulin and low heregulin had significantly higher objective response rate at 58% and significantly longer progression-free survival of 216 days. This result was confirmed in the validation cohort.

**Conclusion:**

A subgroup of CRC patients with high amphiregulin and low heregulin respond to cetuximab therapy better than other patients.

## Introduction

Colorectal cancers (CRC) frequently overexpress epidermal growth factor receptor (EGFR), which is associated with tumor progression and poor prognosis [1]. Thus, EGFR is a therapeutic target, not just against CRC, but also against other cancers in which it is abundantly expressed. Therapeutic agents that target EGFR are either EGFR-tyrosine kinase inhibitors or monoclonal antibodies [2]. Kinase inhibitors such as gefitinib and erlotinib are highly effective against non-small cell lung cancers with constitutively active EGFR mutations [3]. On the other hand, monoclonal antibodies such as cetuximab and panitumumab improve the prognosis in patients with CRC and head and neck squamous cell carcinoma that express wild type EGFR [4–7].

Preclinical studies show that some cancers sensitive to anti-EGFR therapy also abundantly express EGFR ligands, especially amphiregulin [8]. As a result, EGFR is constitutively activated in these cells by autocrine fashion. Notably, patients with CRCs that abundantly express amphiregulin experience significantly better outcomes with cetuximab therapy than patients with low CRC expression of amphiregulin [9, 10]. Indeed, levels of amphiregulin in the plasma are associated with cetuximab efficacy [11].

Heregulin, also ligand of HER3, is also highly expressed in some CRCs, and in some lung, head, and neck cancers [12–15]. Physiologically, heregulin binds to HER3 and triggers heterodimerization between HER2 and HER3, an event that activates both receptors [16, 17]. In turn, HER3 activates AKT, and thereby prevents apoptosis. Published data show that overexpression of heregulin causes cetuximab resistance in CRC, although simultaneous inhibition of HER2 and HER3 overcomes this resistance [13, 18]. In addition, plasma heregulin is negatively correlated with progression free-survival and overall survival in CRC patients undergoing cetuximab therapy.

Clearly, amphiregulin and heregulin significantly impact the prognosis of CRC patients treated with cetuximab. However, clinical outcomes are not always explained by one factor or the other. Some CRC patients abundantly expressing amphiregulin, as well as others with low levels of heregulin, do not respond to cetuximab [11, 13]. A possible reason might be that these molecules interact. Indeed, previous studies demonstrate that while the CRC cell line DiFi abundantly expresses amphiregulin and is sensitive to cetuximab, stable transfection with heregulin causes resistance [18]. Therefore, we examined whether the combined level of circulating amphiregulin and heregulin could more reliably predict clinical outcomes of cetuximab therapy in CRC patients.

## Materials and Methods

### Patients and treatment

The study included patients treated for metastatic CRC at Kinki University School of Medicine between September 2010 and August 2015. Patients had received FOLFIRI (leucovorin, 5-fluorouracil, and irinotecan) or FOLFOX (leucovorin, 5-fluorouracil, and oxaliplatin) as first- or second-line chemotherapy. Most patients had additionally received bevacizumab, but not antibodies against EGFR. As third-line chemotherapy, patients were treated every two weeks with cetuximab, alone or in combination with irinotecan. Unless adjusted by the attending physician, cetuximab was administered at an initial dose of 400 mg/m^2^ and then at 250 mg/m^2^ weekly. The study was approved by the Institutional Review Board of Kinki University School of Medicine. Written informed consent was obtained from all patients. Patients were divided into a training cohort of 50, and a validation cohort of 10.

### Measurement of plasma amphiregulin and heregulin

Plasma samples were drawn from patients prior to treatment with anti-EGFR. Amphiregulin and heregulin levels were measured using commercially available enzyme-linked immunosorbent assay kits (Human Amphiregulin and Heregulin Quantikine ELISA Kits, R&D Systems, Minneapolis, MN, USA), following the manufacturer’s instructions. Briefly, a 96-well microplate was coated with capture antibody, washed, and incubated with samples and standards. The plate was washed a second time, probed with detection antibody, and labeled with a chromogen. Finally, absorbance at 450 nm was measured using a spectrophotometric plate reader. Amphiregulin and heregulin concentrations were determined based on standard curves.

### Assessment of cetuximab treatment

Objective response to cetuximab was evaluated according to the Response Evaluation Criteria in Solid Tumors, ver. 1.1 [19]. Tumor response was evaluated by a physician every 2 to 3 months using computed tomography. Progression-free survival was defined as the interval from the initiation of anti-EGFR therapy to tumor progression or death without evidence of progression. Overall survival was measured as the number of days between the start of cetuximab regimen and death.

### Statistical analyses

The primary endpoint of this study is to evaluate amphiregulin and heregulin as biomarkers associated with objective response rate to anti-EGFR therapy. ROC (receiver operating characteristics) curves were analyzed to determine cut-off points for amphiregulin and heregulin expression. The optimum cut-off points were defined as the minimum distance from (1 –specificity, sensitivity) = (0, 1). Differences in the distribution of variables were evaluated using *t* test for quantitative variables and Fisher’s exact test for qualitative variables. For groups classified according to cut-off points, objective response rate was compared by Fisher’s exact test, while progression-free and overall survival were compared by Kaplan-Meier curves and log-rank test. All statistical tests were two-sided, and a *p* value < 0.05 was considered statistically significant. Statistical analyses and visualization were performed in GraphPad Prism v.5.0 for Windows (GraphPad Software, Inc., La Jolla, CA, USA).

## Results

### Patient characteristics

Patient characteristics are summarized in Table 1. All patients had wild-type *KRAS*. Of the 50 patients analyzed, 39 received cetuximab and irinotecan as third-line chemotherapy. However, all other patients received cetuximab alone because of poor general health or fluid retention. Thirteen patients experienced partial response to cetuximab. Thus, the overall response rate was 26%. Eighteen patients had stable tumors for more than 8 weeks, while 19 patients had short-term cancer progression. The overall disease control rate was 62%. Median progression-free and overall survival were 110 days and 201 days, respectively. Treatment was discontinued for all patients because of cancer progression, except for three patients who moved to a different hospital. Toxicity was not apparent. There were no significant differences in response rate when patients were stratified by for each variable, including age, sex, primary tumor site, ECOG PS, and regimen (Fisher’s exact test, *p* > 0.05, S1 Table).

**Table 1:** Patient characteristics.

Parameter		Values
Age	Median (range)	62 (41–85)
Sex	Male (%)	31 (62)
	Female (%)	19 (38)
Primary tumor site	Colon (%)	31 (62)
	Rectum (%)	19 (38)
ECOG PS	1 (%)	45 (90)
	2 (%)	5 (10)
Regimen	Cetuximab	11 (22)
	Cetuximab + CPT-11	39 (78)
Responses	PR (%)	13 (26)
	SD (%)	18 (36)
	PD (%)	19 (38)

ECOG PS, Eastern Cooperative Oncology Group Performance Status

PR, partial response; SD, stable disease; PD, progressive disease.

### Circulating amphiregulin and heregulin

**Fig 1:**
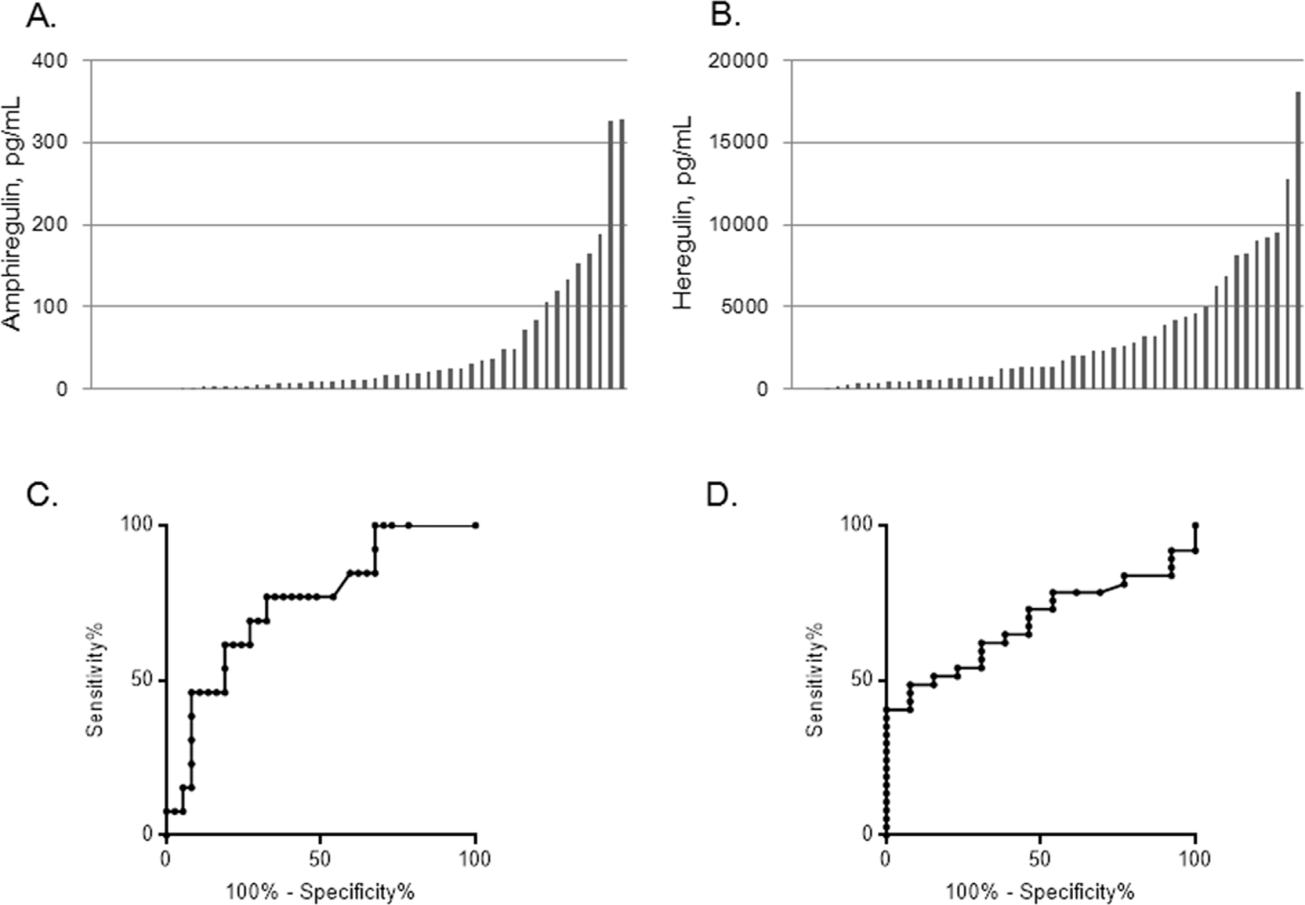
Circulating amphiregulin and heregulin in 50 patients with colorectal cancer. Plasma amphiregulin (A) and heregulin (B) were measured by enzyme-linked immunosorbent assay. Each bar represents a single patient. Receiver operating characteristic (ROC) curves are shown for the ability of amphiregulin (C) and heregulin (D) to predict response and non-response to cetuximab.

Amphiregulin and heregulin concentrations in the plasma are shown in Fig 1A and 1B, with median 11.5 pg/mL (range 0–327 pg/mL) and 1,357.5 pg/mL (range 0–18,045 pg/mL), respectively. Correlation between the concentrations of these molecules was not observed (Pearson correlation r = 0.067), and levels were not significantly correlated with patient characteristics including sex, age, primary tumor site, performance status, and treatment regimen (Table 2, *t* test, p > 0.05). Amphiregulin level tends to be higher in cetuximab-responsive patients, while heregulin level is significant lower in resistant patients (*t* test, *p* = 0.001). Thus, we performed ROC analyses to determine cutoff points at which amphiregulin and heregulin have optimum sensitivity and specificity to predict response or non-response to cetuximab, respectively (Fig 1C and 1D). At 16.8 pg/mL, amphiregulin had sensitivity 76.9% (95% CI: 46.2%–95.0%) and specificity 67.6% (95% CI: 50.2%–82.0%). Using this cutoff point, 22 patients (44%) were considered amphiregulin positive for predicting response with response rate 45% (10/22). On the other hand, heregulin expression at 1292 pg/mL had sensitivity 62.2% (95% CI: 44.8%–77.5%) and specificity 69.2% (95% CI: 38.6%–90.9%). Based on this cutoff point, 27 patients (54%) were considered heregulin positive for predicting non-response with response rate is 15% (4/27).

**Table 2:** Plasma amphiregulin and heregulin stratified by patient characteristics.

Parameter		Amphiregulin median (range), pg/ml	Heregulin median (range), pg/ml
Age	< 70	11.5 (0–188.8)	1,849 (0–18,045)
	> 70	12.9 (0–327.6)	676 (0–9,490)
Sex	Male	10.6 (0–326.4)	1,265 (0–18,045)
	Female	15.9 (0–327.6)	2,294 (0–12,811)
Primary tumor site	Colon	8.2 (0–327.6)	1,371 (0–18,045)
	Rectum	12.9 (2.4–188.8)	1,300 (0–9,180)
ECOG PS	1	9.4 (0–188.8)	1,300 (0–18,045)
	2	21.8 (11.2–327.6)	2,625 (507–8,984)
Regimen	Cetuximab + CPT-11	8.2 (0–188.8)	1,300 (0–18,045)
	Cetuximab	21.8 (1.2–327.6)	2,510 (49–9,490)

ECOG PS, Eastern Cooperative Oncology Group Performance Status; CPT-11, irinotecan.

### Association of amphiregulin and heregulin with cetuximab response

**Fig 2:**
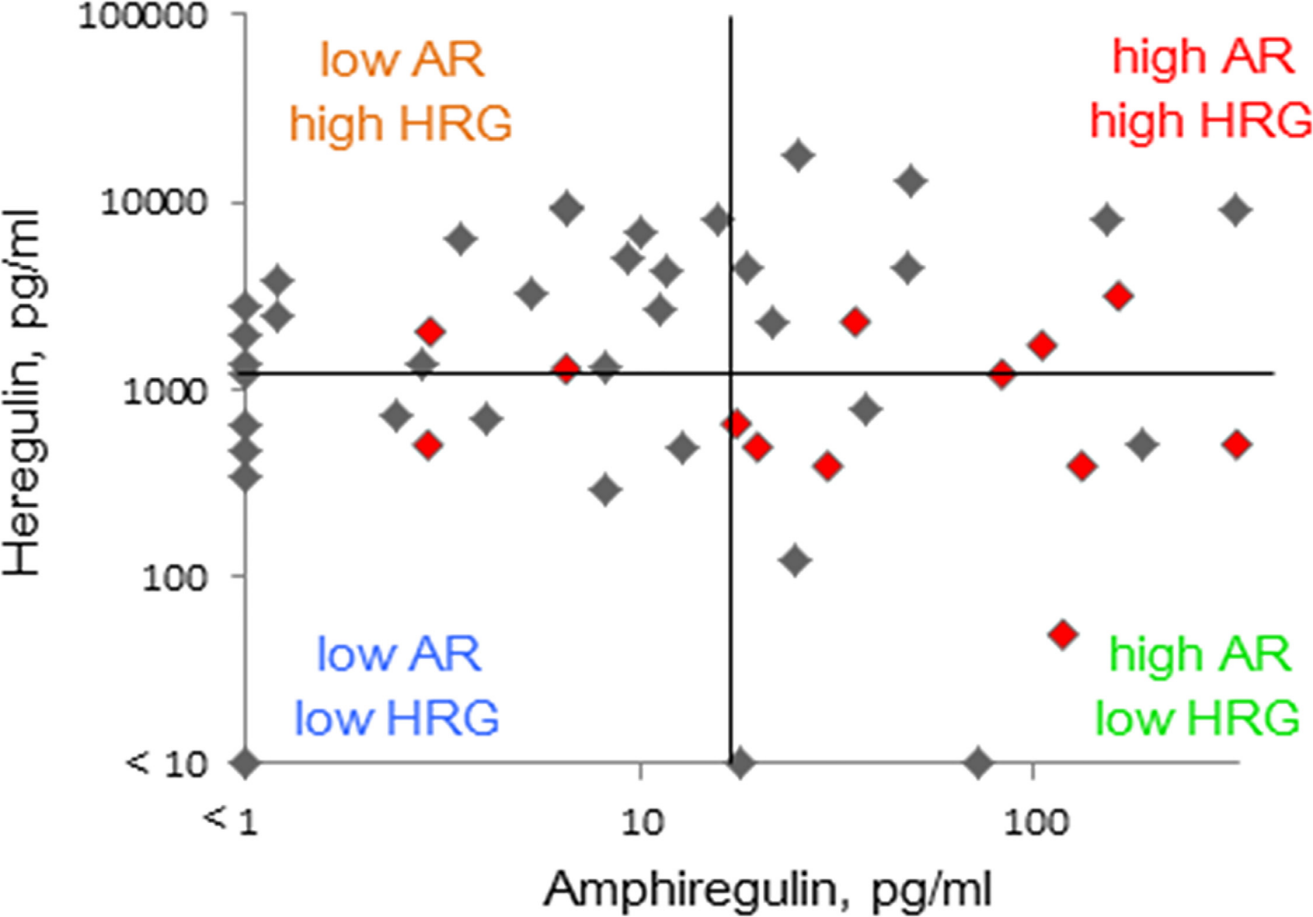
Scatter diagram of plasma amphiregulin and heregulin in 50 patients with colorectal cancer. Patients were categorized into four groups based on median levels of amphiregulin (16.8 pg/mL) or heregulin (1,292 pg/mL). Red dots are patients who responded to anti-EGFR therapy.

We investigated whether the combined level of plasma amphiregulin and heregulin was associated with response to anti-EGFR therapy (Table 3). We categorized patients into four classes depending on whether expression of each molecule was high or low, with cutoff values set by ROC analysis (Fig 2). Thus, 15 patients were considered to have low amphiregulin and high heregulin, while 13 were considered to have low levels of both. Of the remaining patients, 10 were categorized as having high levels of both, and 12 were described as having high amphiregulin and low heregulin. Objective response rate was evaluated in each class. In patients that abundantly express amphiregulin but not heregulin, the objective response rate was 58%. Notably, this response rate is significantly different (*p* = 0.0069, Fisher’s exact test) from the 16% response rate in other patients (n = 38). Furthermore, the response rate was 7% in patients with low amphiregulin and high heregulin, 15% in patients with low amphiregulin and low heregulin, and 30% in patients that express both ligands abundantly.

**Table 3:** Objective response rate.

Heregulin	Amphiregulin	
	Low	High
**Low**	15%	58%
**High**	7%	30%

### Association of plasma amphiregulin and heregulin with survival

**Fig 3:**
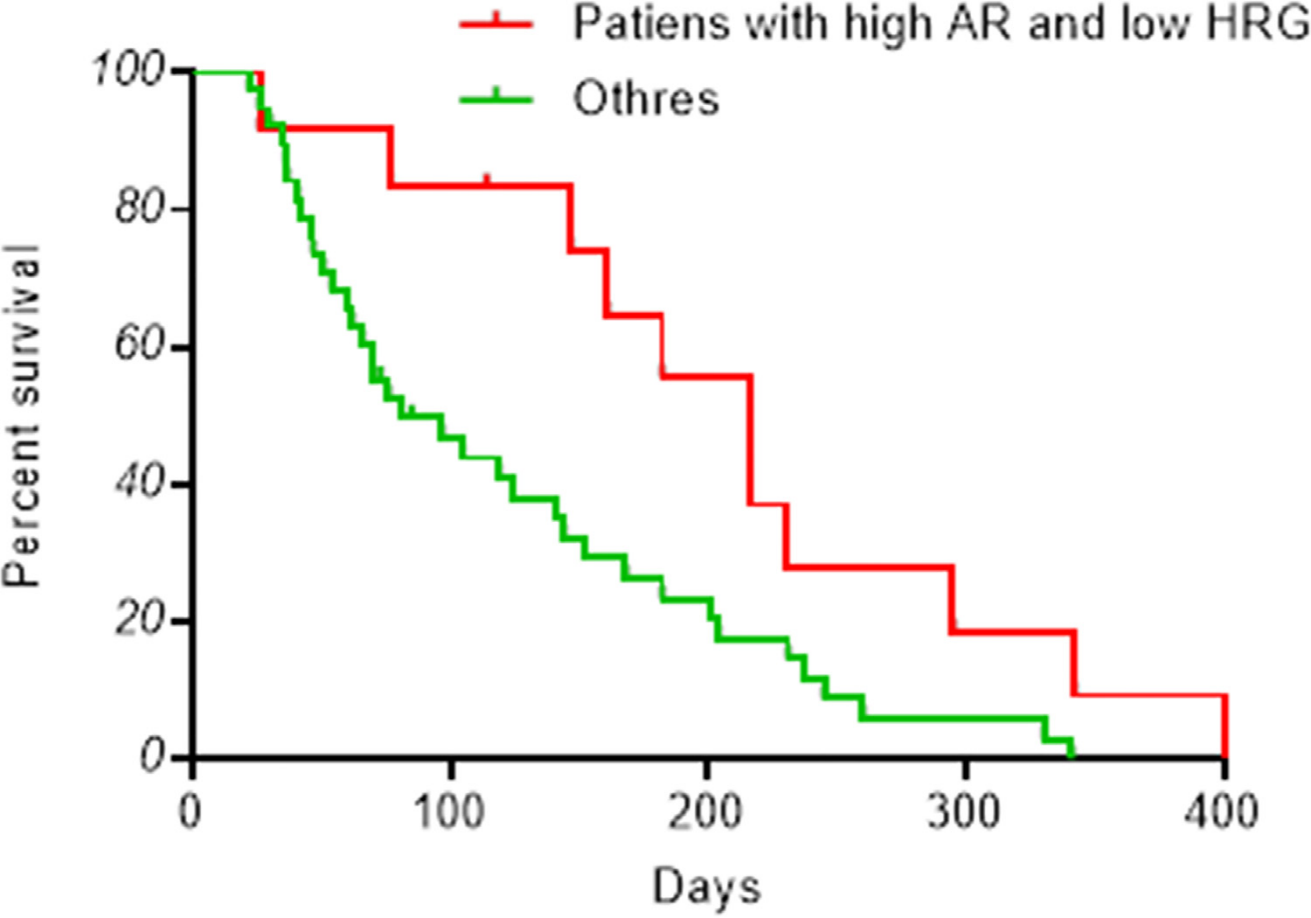
Kaplan-Meier progression-free survival curves. Plasma levels of amphiregulin and heregulin were measured by ELISA. Patients are divided into subgroups based on amphiregulin (AR) or heregulin (HRG) abundance, as shown in Fig 2. The red curve represents survival in patients with high amphiregulin and low heregulin (n = 12), and the green line represents survival in all other patients (n = 38).

We analyzed the impact of amphiregulin and heregulin levels on progression-free and overall survival. Consistent with better response rates, patients who abundantly express amphiregulin but not heregulin experienced significantly longer progression-free survival than other patients (log-rank test, *p* < 0.05), with median 216 days and 81 days, respectively (Fig 3). Patients that express both molecules at high levels experienced a median of 65 progression-free days, while those that express both at low levels had median progression-free survival of 119 days. Finally, patients who have low amphiregulin and high heregulin had median 76 progression-free days (Fig A in S1 File).

**Fig 4:**
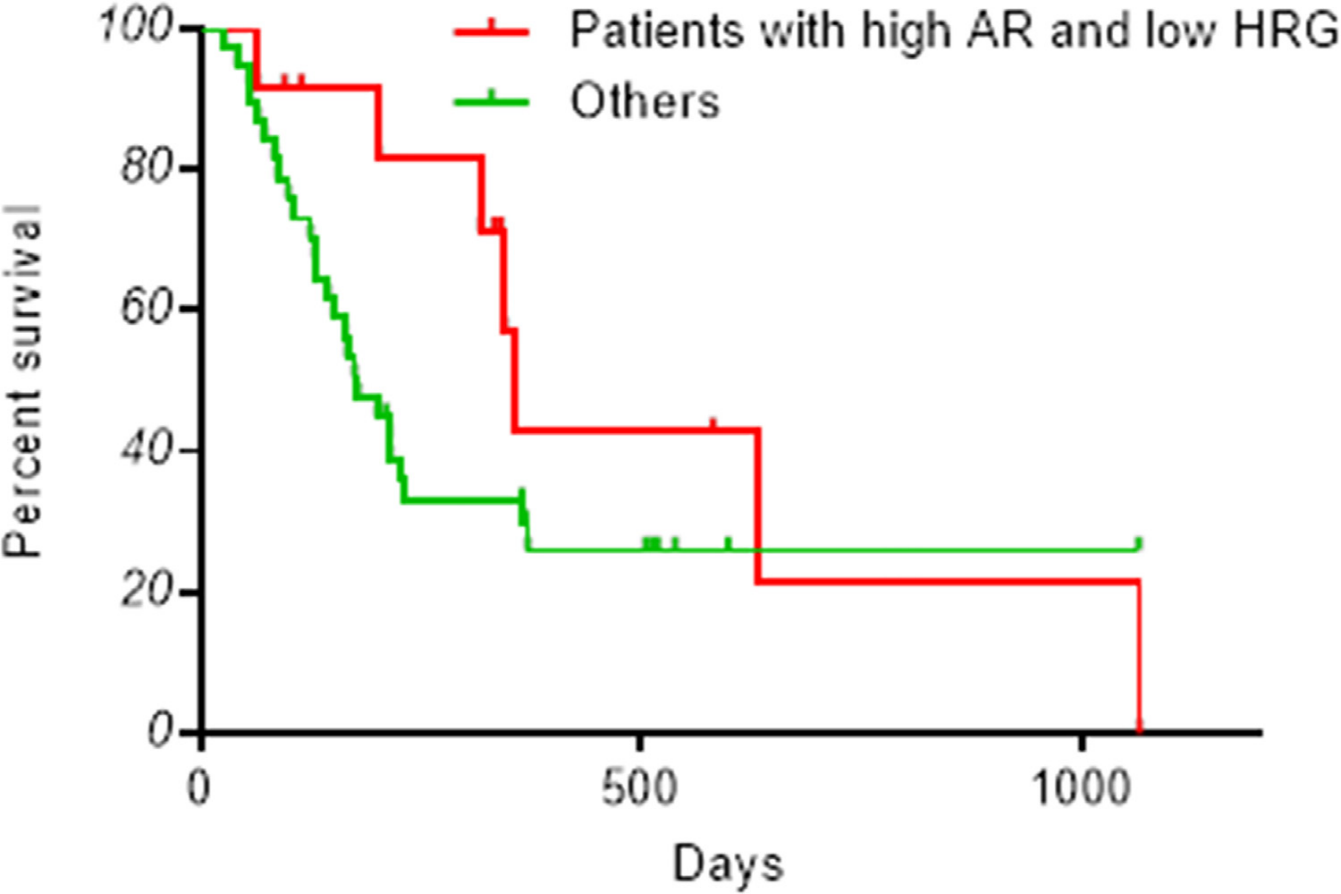
Kaplan-Meier overall survival curves. Plasma levels of amphiregulin and heregulin were measured by ELISA. Patients are divided into subgroups based on amphiregulin (AR) or heregulin (HRG) abundance, as shown in Fig 2. Survival in patients with high amphiregulin and low heregulin (n = 12) is plotted in red. Survival in all other patients (n = 38) is plotted in green.

These trends are reflected in overall survival. Thus, patients with high levels of amphiregulin and low levels of heregulin tended to experience longer overall survival than other patients with median 356 days (Fig 4). Those with low or high levels of both molecules survived for a median of 231 and 164 days, respectively. Finally, patients who express heregulin abundantly but not amphiregulin had median overall survival of 150 days (Fig B in S1 File).

### Plasma amphiregulin, heregulin, and cetuximab response in a validation cohort

We validated the association of both ligands with cetuximab response using an independent set of 10 patients. Three of these patients had high amphiregulin and low heregulin, while three had low amphiregulin and high heregulin. Of the remaining patients, three expressed both ligands at low levels, and one expressed both abundantly (Fig C in S1 File). Three patients were responsive to anti-EGFR therapy, all of whom had high amphiregulin but not heregulin (Table 4).

**Table 4:** Objective response rate in the validation cohort.

Heregulin	Amphiregulin	
	Low	High
**Low**	0% (0/3)	100% (3/3)
**High**	0% (0/3)	0% (0/1)

## Discussion

We and others have previously reported that EGFR ligands, especially amphiregulin, are associated with cetuximab efficacy [8–10]. In contrast, the HER3 ligand heregulin was significantly correlated with cetuximab resistance [13, 18]. This study is the first to examine whether the combined expression of amphiregulin and heregulin affects the outcome of anti-EGFR therapy in CRC patients. We found that patients with high levels of amphiregulin and low levels of heregulin respond significantly better to cetuximab, with 58% objective response rate and 216 days median progression-free survival. The response is notably better than the published overall response in the general CRC population [4, 20, 21]. These results were validated in a smaller, but independent cohort, and suggest that assessment of both amphiregulin and heregulin in the plasma may more precisely predict cetuximab outcome, and may thus provide a basis for optimizing CRC treatments.

In contrast, patients that abundantly express both amphiregulin and heregulin experience poor outcomes. This clinical observation is consistent with laboratory experiments using the CRC cell line DiFi [18]. Indeed, DiFi cells with high levels of amphiregulin have increased sensitivity to cetuximab, whereas cell overexpressing heregulin are resistant to cetuximab *in vitro* and in mouse xenografts. Therefore, patients with these characteristics may require additional, new, or alternative treatments to overcome resistance. For example, it has been demonstrated that cells overexpressing heregulin are sensitive to a combination of cetuximab and patritumab, an antibody against HER3, even though each antibody on its own is ineffective [18]. Alternatively, combining cetuximab with a downstream inhibitor of HER3 may prove also prove effective. Such compounds include inhibitors of PI3K and AKT.

Notably, three patients with low levels of amphiregulin also responded to cetixumab, suggesting that EGFR is activated by other ligands in a small group of patients. Indeed, EGFR is known to bind six different ligands, including EGF, transforming growth factor-α, amphiregulin, betacelluline, heparin-binding EGF, or epiregulin [8]. Of these, epiregulin expression in tumors has been found to also correlate with cetuximab efficacy [9, 10]. In the end, all EGFR ligands may have to be evaluated to predict cetuximab response.

However, this study is limited by small sample sizes, and the retrospective nature of the analysis. To address these limitations, we are now conducting a prospective randomized clinical trial of anti-EGFR therapy in advanced CRC patients, in which we measure clinical outcome as a function of plasma amphiregulin and heregulin. We anticipate that this trial will provide more evidence of the use of amphiregulin and heregulin as biomarkers.

## Supporting Information

S1 Dataset Full data set. (XLSX)

S1 File Kaplan-Meier progression-free survival curves (Fig A). Kaplan-Meier overall survival curves (Fig B). Scatter diagram of plasma amphiregulin and heregulin in validating cohort including 10 patients with colorectal cancer (Fig C). (PDF)

S1 Table Response rate when patients were stratified by for each variable. (PPTX)
