# Supplementary material for: Combined Analysis of Plasma Amphiregulin and Heregulin Predicts Response to Cetuximab in Metastatic Colorectal Cancer
Source: PLoS One. 2015 Nov 16;10(11):e0143132. doi: 10.1371/journal.pone.0143132 (PMC4646631; doi:10.1371/journal.pone.0143132)
Supplement: S1 File — (PDF) [file pone.0143132.s002.pdf]

Figure A

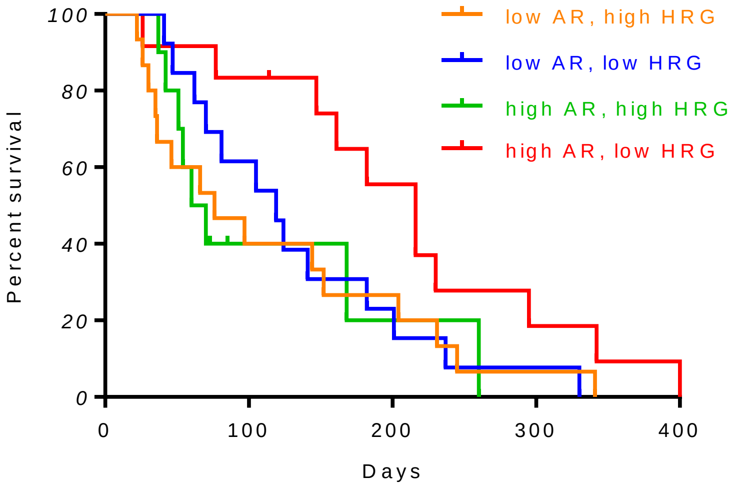

Figure A. Kaplan-Meier progression-free survival curves.
Plasma levels of amphiregulin and heregulin were measured by ELISA. Patients were divided into four groups based on amphiregulin or heregulin abundance, as shown in Supplement Figure 1. AR, amphiregulin; HRG, heregulin.

Figure B

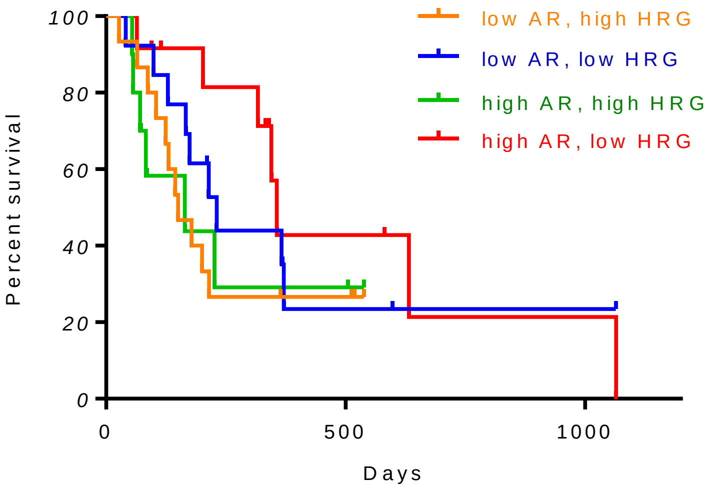

Figure B. Kaplan-Meier overall survival curves.
Plasma levels of amphiregulin and heregulin were measured by ELISA. Patients were divided into four groups based on amphiregulin or heregulin abundance, as shown in Supplement Figure 2. AR, amphiregulin; HRG, heregulin.

Figure C

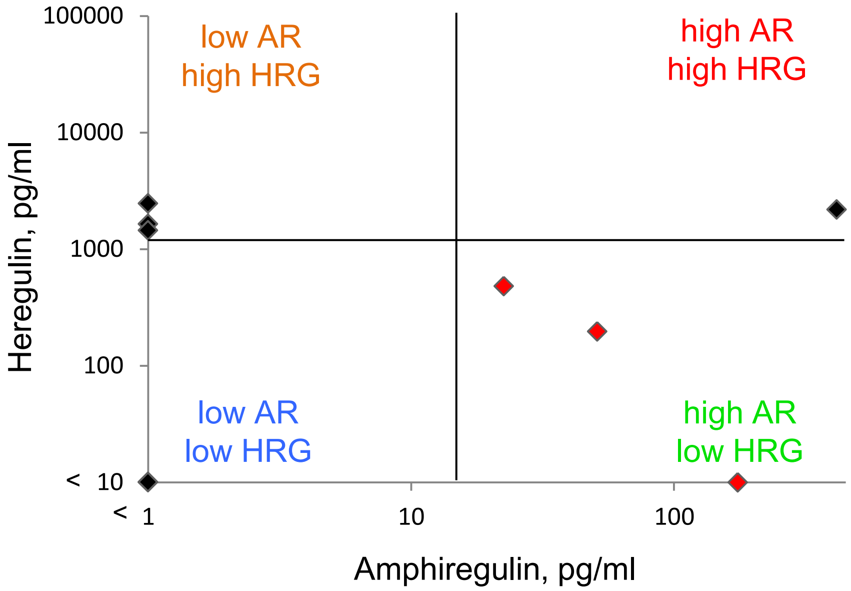

Figure C. Scatter diagram of plasma amphiregulin and heregulin in validating cohort including 10 patients with colorectal cancer.
Patients were categorized into four groups based on median levels of amphiregulin or heregulin, 16.8pg/ml and 1292pg/ml, respectively. Red dot shows a patient, who achieved response with anti-EGFR.
