# Supplementary material for: Combined Analysis of Plasma Amphiregulin and Heregulin Predicts Response to Cetuximab in Metastatic Colorectal Cancer
Source: PLoS One. 2015 Nov 16;10(11):e0143132. doi: 10.1371/journal.pone.0143132 (PMC4646631; doi:10.1371/journal.pone.0143132)
Supplement: S1 Table — (PPTX) [file pone.0143132.s003.pptx]

## Slide 1
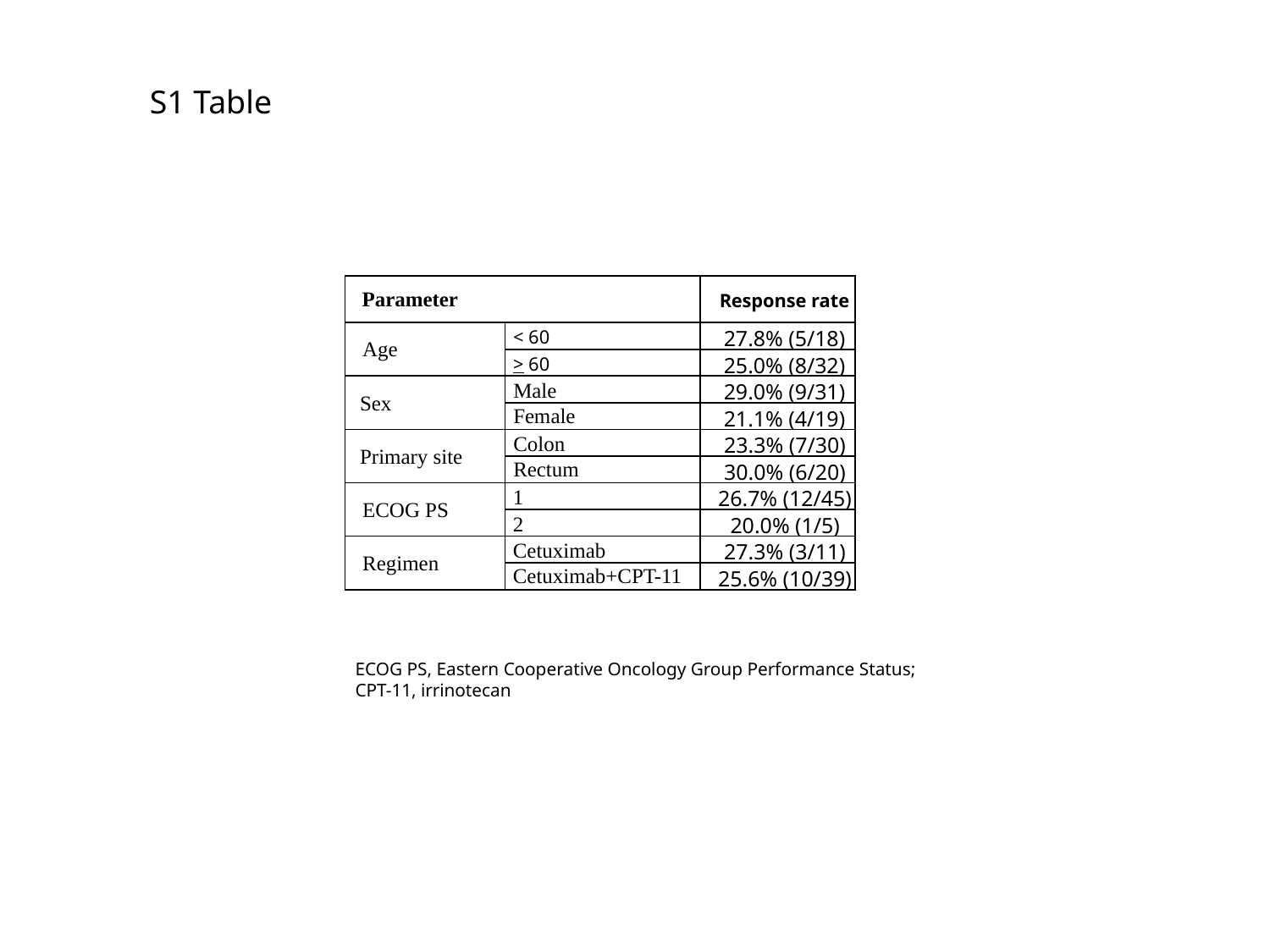

S1 Table
| Parameter | | Response rate |
| --- | --- | --- |
| Age | < 60 | 27.8% (5/18) |
| | > 60 | 25.0% (8/32) |
| Sex | Male | 29.0% (9/31) |
| | Female | 21.1% (4/19) |
| Primary site | Colon | 23.3% (7/30) |
| | Rectum | 30.0% (6/20) |
| ECOG PS | 1 | 26.7% (12/45) |
| | 2 | 20.0% (1/5) |
| Regimen | Cetuximab | 27.3% (3/11) |
| | Cetuximab+CPT-11 | 25.6% (10/39) |
ECOG PS, Eastern Cooperative Oncology Group Performance Status;
CPT-11, irrinotecan
